# Evaluation of the Performance of Bio-Based Rigid Polyurethane Foam with High Amounts of Sunflower Press Cake Particles

**DOI:** 10.3390/ma14195475

**Published:** 2021-09-22

**Authors:** Agnė Kairytė, Sylwia Członka, Renata Boris, Sigitas Vėjelis

**Affiliations:** 1 Laboratory of Thermal Insulating Materials and Acoustics, Faculty of Civil Engineering, Institute of Building Materials, Vilnius Gediminas Technical University, Linkmenu Str. 28, LT-08217 Vilnius, Lithuania; sigitas.vejelis@vilniustech.lt; 2 Institute of Polymer & Dye Technology, Lodz University of Technology, 90-924 Lodz, Poland; sylwia.czlonka@dokt.p.lodz.pl; 3 Laboratory of Composite Materials, Faculty of Civil Engineering, Institute of Building Materials, Vilnius Gediminas Technical University, Linkmenu Str. 28, LT-08217 Vilnius, Lithuania; renata.boris@vilniustech.lt

**Keywords:** sunflower press cake, polyurethane foam, thermal insulation, sustainability, mechanical performance

## Abstract

In the current study, rigid polyurethane foam (PUR) was modified with 10–30 wt.% sunflower press cake (SFP) filler, and its effect on performance characteristics—i.e., rheology, characteristic foaming times, apparent density, thermal conductivity, compressive strength parallel and perpendicular to the foaming directions, tensile strength, and short-term water absorption by partial immersion—was evaluated. Microstructural and statistical analyses were implemented as well. During the study, it was determined that 10–20 wt.% SFP filler showed the greatest positive impact. For instance, the thermal conductivity value improved by 9% and 17%, respectively, while mechanical performance, i.e., compressive strength, increased by 11% and 28% in the perpendicular direction and by 43% and 67% in the parallel direction. Moreover, tensile strength showed 49% and 61% increments, respectively, at 10 wt.% and 20 wt.% SFP filler. Most importantly, SFP filler-modified PUR foams were characterised by two times lower water absorption values and improved microstructures with a reduced average cell size and increased content in closed cells.

## 1. Introduction

It is estimated that within 10 years, global energy consumption will increase by ~50% [1]. In order to reduce energy demands and greenhouse gas emissions, the easiest way to do so is through thermal insulation of buildings. If suitably selected and installed, thermal insulating layers can save energy for heating in winter and cooling in summer. 

Rigid polyurethane foams are characterised by the lowest thermal conductivity, which varies from 0.018 W/(m·K) to 0.025 W/(m·K) when the traditional blowing agents are used, and from 0.033 W/(m·K) to 0.040 W/(m·K) when water is used as a chemical blowing agent [2]. Therefore, this building material has becomes an extremely important cellular plastic due to its versatility and the possibility of its application in various industries such as those concerned with thermal insulating layers, bedding, furniture, automotive seats and everywhere where other materials might not be suitable [3]. 

Polyurethane foams can be produced from mass polymerisation, which is the easiest technique for polyurethane foam synthesis and solely uses monomers such as polyols, isocyanates and physical or chemical blowing agents [4]. Currently, these main raw materials have become unpopular because they are based on petrochemical resources and this has prompted the polyurethane industry and scientific community to develop greener raw materials such as polyols from various natural or waste plant oils [5,6,7], and isocyanates from fatty or amino acids [8], while ozone depletion and global warming have caused the development of blowing agents based on hydrofluoroolefins and water [9,10] and the implementation of natural resources as fillers in the form of fibres [11,12] or particulates [13,14]. The application of these sustainable raw materials contributes to the reduction of greenhouse gas emissions, increased environmental conservation, stability and sustainability, and promotes convenient and cost-efficient ways to produce bio-based polyurethane composites.

Currently, there are several studies on the use of natural fillers in polyurethane foam composites. For instance, the authors of [15] investigated the effect of coconut husk and corn cob fillers on the apparent density, mechanical performance, and thermal conductivity of polyurethane foam. To demonstrate the impact of these fillers, they varied the amount of fillers from 5% to 25% and noted that the apparent density, thermal conductivity, and compression set improved, while tensile strength and elongation at break had significant reductions. However, another natural filler—nutmeg particles at amounts of (1–5%) incorporated into polyurethane foam—revealed that mechanical performance increased only up to a certain limit, i.e., up to 2% of nutmeg filler, and thermal conductivity deteriorated at all amounts of filler, although fire resistance, and antibacterial and anti-ageing properties of modified polyurethane foams were improved [16]. Disregarding the positive or negative impacts on apparent density and compression set or compressive strength of natural filler-reinforced polyurethane foams, the study [17] showed that the addition of hazelnut and walnut shells improved the thermal stability of the resulting products, while [18] revealed that chemically modified lignin used as a natural filler in polyurethane foams decreased thermal degradation at low temperatures but increased it at higher ones. Moreover, [19] highlighted that 2% of sunflower press cake particles (SFP) improved the thermal stability of polyurethane foam at all degradation stages but increased thermal conductivity by 4% and compressive strength and flexural strength by 8% and 3%, respectively. The authors thought it would be an interesting idea for press cakes to be used as natural fillers for the modification of polyurethane foams, since SFP is a by-product of sunflower oil production and it contains components which are insoluble in water. According to Ancuţa and Sonia [20], press cakes can be used to feed animals because they are rich in proteins, cellulose, and hemicellulose. Regrettably, due to antinutritional factors such as phytic acid and trypsin inhibitors, press cakes may negatively affect the health of animals and humans. Another problem is landfilling, during which waste decomposes, thus inducing the production of methane and the pollution of water. Furthermore, press cakes can be converted into energy through incineration. However, it is not cost-effective and negatively impacts environmental emissions.

The aim of this study is to investigate and evaluate the impact of high amounts of crushed SFP on rigid polyurethane (PUR) foam obtained from rapeseed oil- and sucrose-based polyols and water as a blowing agent. Moreover, rheological and structural characterisation, foaming process evaluation, and testing of thermal insulation, water resistance and mechanical performance were conducted, and the statistical interpretation of the results was implemented.

## 2. Materials and Methods

Polyol BioPolyol RD was obtained from rapeseed oil (SIA PolyLabs, Riga, Latvia) and Petol PZ 400-4G from sucrose (Oltchim, Râmnicu Vâlcea, Romania). They were used as the main components for the production of PUR foam and SFP filler-modified PUR foams. Polyol from rapeseed oil has the hydroxyl value of 350 mg KOH/g, while the sucrose-based oil has a value of 421 mg KOH/g. Water contents of each polyol were <0.2% and <0.1%, respectively. As a second main component, polymeric 4,4′ -diphenylmethane diisocyanate Lupranate M20S (BASF, Ludwigshafen, Germany) was selected. All PUR foams, including SFP filler-modified PUR foams, were blown using distilled water. To catalyse blowing and gelling reactions, Polycat 9 (Air Products and Chemicals, Inc., Allentown, PA, USA) was incorporated. To stabilise foams and form cellular structures, silicone surfactant ST-52 (Shijiazhuang Chuanghong Technology Co., Ltd., Shijiazhuang, China) was added. The SFP filler was supplied by a local company (Vilnius, Lithuania).

**Figure 1:**
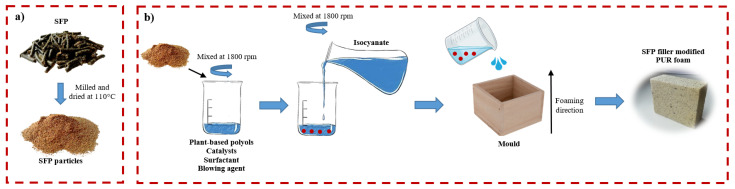
Graphical representation of the preparation scheme of: (**a**) SFP filler and (**b**) SFP filler-modified PUR foams.

The selected amounts of raw materials used for the preparation of PUR foam and SFP filler-modified PUR foams are presented in Table 1 and the graphical preparation scheme in Figure 1. The PUR foam and SFP filler-modified PUR foams were produced by a two-component system which had an equivalent ratio of isocyanate and hydroxyl groups equal to 1.25:1. As 100% rapeseed oil-based polyol does not produce dimensionally stable foams, its amount was reduced to 40%. In order to obtain stable PUR foams, 60% of sucrose-based polyol was incorporated.

The system of two polyols was mixed with water, catalyst, and surfactant, and thoroughly mixed at a speed of 1800 rpm for 1 min. Before the addition of SFP filler, it was crushed and milled with a laboratory shredder for 1 min and thermally treated at a temperature of 110 °C for 24 h to remove the excess of moisture. After that, 10 wt.%, 20 wt.%, and 30 wt.% of SFP filler were added into the prepared mixture. The obtained premix was thoroughly mixed with isocyanate for 10 s at 1800 rpm. The obtained viscous mass was immediately poured into moulds and left to cure at a temperature of (23 ± 2) °C.

The moisture content of particles was determined after drying three samples at (105 ± 2) °C until the change in mass between three weighings made 24 h apart was less than 0.1%, in accordance with the requirements outlined by [21]. 

The structural studies of SFP filler, neat PUR foam and SFP filler-modified PUR foams were conducted using scanning electron microscopy (SEM) JEOL SM–7600F (JEOL Ltd., Tokyo, Japan). Before the analysis of the samples, the filler and foams were coated with a gold layer under vacuum with the QUORUM Q150R ES (Quorum Technologies Ltd., Lewes, UK) apparatus. The obtained images were used to further analyse structural parameters such as average cell size and the size of filler particles with ImageJ software.

The test of dynamic viscosity was made using an SV-10 viscometer (A&D Company Ltd., Tokyo, Japan), which has an accuracy of 0.01 mPa·s and a measuring range of up to 1200 mPa·s.

The characteristic foaming times and reaction profile were determined according to [22], Annex E. Before the test, all components were conditioned at a temperature of (20 ± 1) °C. The measurements were conducted using an electronic stopwatch with an accuracy of 0.5 s.

The linear dimensions of the samples were determined based on the requirements of [23], while the apparent density was determined according to [24] for 50 × 50 × 50 mm-sized samples. 

Thermal conductivity measurements were conducted for 300 × 300 × 50 mm-sized samples, according to [25], with a FOX 304 (TA Instruments, United States of America) heat flow meter, which has an active edge insulation. During the test, the direction of heat flow was upwards. 

The determination of the volumetric percentage of closed cells was carried out based on [26], method 2 for 100 × 30 × 30 mm-sized samples.

To determine the impact of water on the neat PUR foam and SFP filler-modified PUR foam, short-term water absorption by partial immersion was chosen according to ([22], Table 7) and carried out according to [27] (officially replacing [28]) for samples having the size of 200 × 200 × 50 mm. After the test, samples were drained for 10 min using a drainage stove.

The compressive strength and tensile strength were obtained in accordance with [29] and [30], respectively for the samples having the size of 50 × 50 × 50 mm with the H10KS (Hounsfield, United Kingdom) universal testing machine.

## 3. Results and Discussion

**Figure 2:**
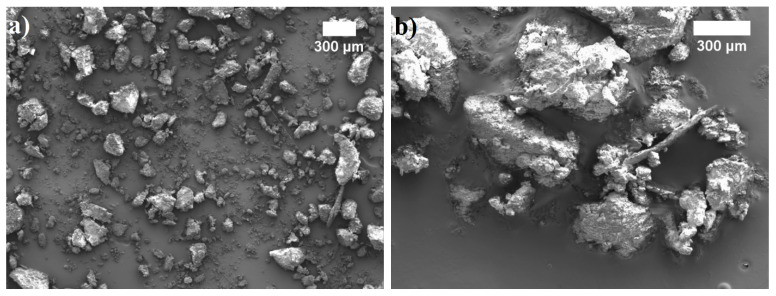
SFP filler: (**a**) at ×40 magnification and (**b**) at ×60 magnification.

Figure 2 presents the external appearance of SFP filler. The microscopic investigation revealed the presence of micron-sized particles with a wide granulometric distribution of 2–695 μm. It can also be observed that the filler particles are not homogeneous; some of them are agglomerated and have a rough surface, which in turn may positively affect the adhesion ability between the SFP filler and polyurethane matrix.

The moisture content of fillers in PUR foam compositions is of great importance due to the mixture’s sensitivity to water and/or moisture during reaction with isocyanates. The parameter is too important to neglect its impact on foaming and apparent density of the final products. Therefore, the experimental investigation showed that the original moisture content of SFP filler particles was 8.4 ± 0.3 wt.% and it confirms the results obtained in other studies [31,32], i.e., up to 11 wt.%. However, after the thermal treatment at 110 °C, the moisture content was reduced to 1.1 ± 0.2 wt.%. According to the results presented in Table 2, the addition of SFP filler greatly impacts the characteristic foaming times of polyurethane mixtures.

Compared to the neat PUR foam, the cream time increases by 24%, 60% and 112%, gel time by 19%, 36% and 68%, and tack-free time by 26%, 47% and 79%, all at 10 wt.%, 20 wt.% and 30 wt.% SFP filler, respectively. It was previously observed by [19] that the presence of hydroxyl groups of SFP filler can interrupt the stoichiometry of synthesis due to the reaction between OH groups of SFP filler and NCO groups of isocyanate. As a result, the higher content of NCO groups is consumed and a reduced amount of CO_2_ is generated, thus slowing down the foaming process. The changes in characteristic foaming times can also be attributed to a higher dynamic viscosity of PUR foam mixtures with a higher content of SFP filler because it impedes the expansion and formation of larger cells. Very similar trends were observed in [33]’s study, where the authors incorporated silanised and non-treated plum stone filler into PUR foam compositions. Although, some natural raw materials such as natural cork have the opposite effect and reduce cream and gel times by 28% and 20%, respectively, and this phenomenon is attributed to the low thermal conductivity of cork agglomerates [34].

Compared to PUR foam without the addition of SFP filler, the incorporation of 10–30 wt.% of SFP increases the dynamic viscosity of the polyol premixes. The highest increase has been noticed in the case of premixes containing 30 wt.% SFP filler. The parameter increases rapidly from 115 mPa·s to 400 mPa·s. The result may be explained by the fact that SFP filler particles tend to agglomerate and form coarse aggregates.

**Figure 3:**
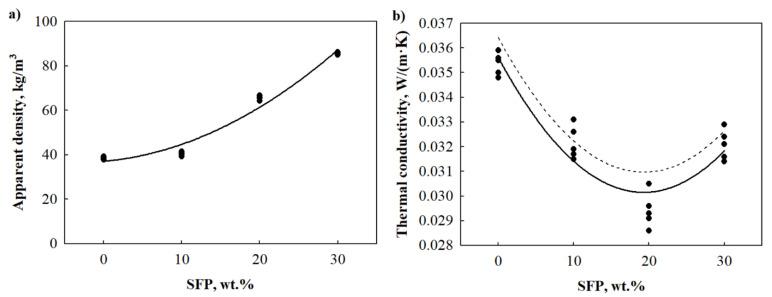
Dependence of properties on the amount of SFP filler: (**a**) apparent density and (**b**) thermal conductivity. •—individual values of thermal conductivity at specified amount of SFP filler; Continuous line—dependence line; Dotted line—upper limit at 90% confidence level.

The addition of SFP filler to PUR foams resulted in an apparent density increase (Figure 3a). From an industrial point of view, modified PUR foams at 10 wt.% SFP filler content have a favourable apparent density value, which is 5% higher compared to the neat PUR foam. However, 20–30 wt.% SFP filler contents lead to a 69% and 120% increase in apparent density. The growth of apparent density values is evident in almost all inorganic and organic filler-modified PUR foams. For instance, [35] reported that the addition of eucalyptus fibre in a range from 8 wt.% to 16 wt.% in PUR foams led to a 10–28% increase in apparent density value. Moreover, [36] obtained a 78% increase in apparent density of PUR foams modified with 20 wt.% of ground tire rubber compared to the neat PUR foam. The magnitude of the increase in apparent density is basically related to filler characteristics and mixture formulations, i.e., the bulk density of the filler, filler content in the premixes, moisture content in the filler and, of course, whether the moisture content is considered while preparing premixes or not, as the extra moisture/water will cause an additional expansion of the foaming mixture.

The change in apparent density of SFP filler-modified PUR foams may also be described by the regression equation (Equation (1)) with determination coefficient $R_{SFP}^{2}=0.973$ and standard deviation $S_{SFP}=3.46$ kg/m^3^.

(1) $$\rho_{SFP}=37.105+0.3115\cdot m_{SFP}+0.04525\cdot m_{SFP}^{2}$$

where $\rho_{SFP}$—apparent density of SFP filler-modified PUR foams, kg/m^3^, and $m_{SFP}$—SFP filler content, wt.%.

Thermal conductivity is one of the most important parameters for thermal insulating materials and is mainly determined by morphological features and apparent density (Table 3). Therefore, it is of great interest to gain knowledge on how natural resources impact the mentioned parameter. It has been reported that the incorporation of various cellulosic fillers in PUR foams increases thermal conductivity [37,38]. However, different observations were conducted with SFP filler-modified PUR foams (Figure 3b). The average value of thermal conductivity for the neat PUR foam is 0.0354 W/(m·K), which is similar to traditional thermal insulating materials such as expanded polystyrene, stone wool, or extruded polystyrene.

The advantage of SFP incorporation can be noticed at 10 wt.% and 20 wt.% contents which reduce thermal conductivity values by 9% and 17%, respectively. Such a result may be explained by the increasing content of closed cells and reduced average cell size (Table 3). Even though SFP filler-modified water-blown PUR foams have higher values of thermal conductivity compared to traditional hydrofluorocarbons-blown PUR foams, it is worth mentioning that the use of plant-based fillers and polyols increases the renewable content of the resultant products while water, which is used as a blowing agent, does not contribute to the depletion of the ozone layer. However, further studies on alternative blowing agents, such as fourth generation olefin-based ones, may result in even lower thermal conductivity values.

**Figure 4:**
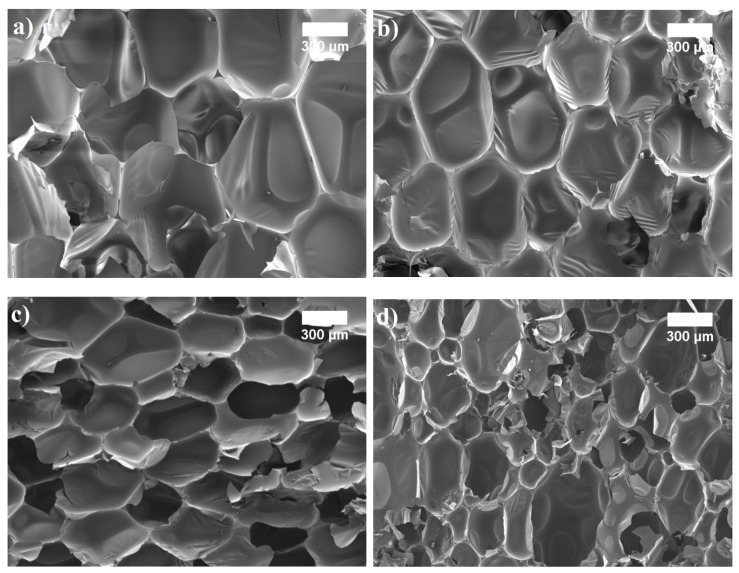
Microstructure of SFP filler modified PUR foams (×50 magnification): (**a**) at 0 wt.%; (**b**) 10 wt.%; (**c**) 20 wt.% and (**d**) 30 wt.%.

Figure 4b and Figure 4c show that the addition of 10–20 wt.% SFP filler leads to a more uniform structure; on average, it increases the content of closed cells from 81 vol.% to 87 vol.%, respectively, and reduces the cell size by 9% and 36% compared to the neat PUR foam. A higher content of closed cells in the case of SFP filler-modified PUR foams can be associated with sufficient interfacial adhesion between the filler surface and the polymer matrix. In addition, the authors of [35] and [39] have concluded that average cell size has a great impact on the total thermal conductivity value of composite materials, and alteration in the cell morphology can also be related to the ability of fillers to act as gas nucleation sites during foaming and their ability to contribute to the formation of nucleating centres for the gaseous phase.

However, 30 wt.% SFP filler started to increase the thermal conductivity value and it became similar to the one at 10 wt.% SFP filler content. Even though the average cell size at such a filler content was reduced by 52% compared to the neat PUR foam, the microstructure (Figure 4d) became non-uniform, and large voids and destruction of the cellular structure were evident. According to [40], this is very common in composite foams because of the increase in viscosity in the prepolymerisation stage, which promotes the cohesion of cells during curing and leads to an increase in thermal conductivity value compared to modified PUR foams with 10–20 wt.% SFP filler contents. The obtained thermal conductivity, closed cell content, and average cell size results show that in all cases SFP filler improves the structure, its parameters, and the overall thermal performance of modified PUR foams.

Furthermore, it is evident that the results of thermal conductivity of modified PUR foams is dependent on the content of SFP filler. Therefore, it is of great importance to evaluate the strength of their impact on the final PUR foams. Test results indicate that the thermal conductivity of SFP filler-modified PUR foams may be approximated by the regression equation (Equation (2)) presented below.

(2) $$\lambda_{SFP}=0.035360-0.000178\cdot m_{SFP}-0.000022\cdot m_{SFP}^{2}+0.000001\cdot m_{SFP}^{3}$$

where $\lambda_{SFP}$—thermal conductivity of SFP filler-modified PUR foams, W/(m·K), and $m_{SFP}$—SFP filler content, wt.%.

The resulting data show that the determination coefficients for the presented equation are $R_{SFP}^{2}=0.936$, the standard deviation—$S_{\lambda_{SFP}}=0.000615$, and Student’s criterion $t_{\alpha }=1.325$ and α=0.90.

The obtained determination coefficients indicate the suitability of the regression model for the proper description of thermal conductivity values of SFP filler-modified PUR foams.

**Figure 5:**
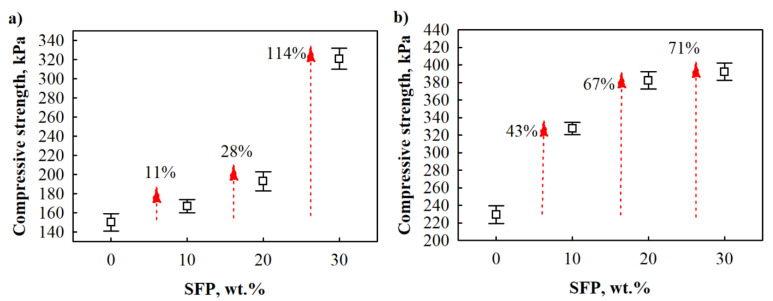
Compressive strength of SFP filler-modified PUR foams: (**a**) perpendicular to foaming direction and (**b**) parallel to foaming direction.

The mechanical performance of polymeric porous materials is influenced by a few factors such as closed cell content, apparent density of the final product, and filler topology, i.e., its ability to effectively adhere to the polymer matrix. Compared to neat PUR foam, SFP filler-modified PUR foams showed a noticeable improvement in compressive strength in the perpendicular and parallel directions (Figure 5). When it came to 10 wt.% SFP, there was a slight increase in the compressive strength in the perpendicular direction, i.e., 11%, while improvement in the parallel direction was 43% compared to unfilled PIR foam. A further increase in SFP filler content to 20 wt.% strengthened PUR foams by 28% and 67% in the perpendicular and parallel directions, respectively. Some studies [41,42,43] have shown that up to 20 wt.% of certain fillers in polyurethane foam systems is the content limit at which the mechanical performance of filled PUR foams improves, and further addition of filler is not favourable. However, contrary observations were conducted at 30 wt.% SFP, which significantly increased the compressive strength in both directions, i.e., 114% in the perpendicular and 71% in the parallel directions. The obtained results indicate great compatibility between SFP filler and PUR matrix. As [44] reported, the addition of natural fillers impacts the crosslink density of PUR foams due to hydroxyl groups of lignin and cellulose. They react with isocyanate groups by forming a more compact structure in PUR foams and increasing the number of urethane groups, which provide additional crosslink sites and increase the number of hard segments. Additionally, the more compact structure between SFP filler and PUR foam matrix assures an efficient stress transfer from matrix to filler during compression.

**Figure 6:**
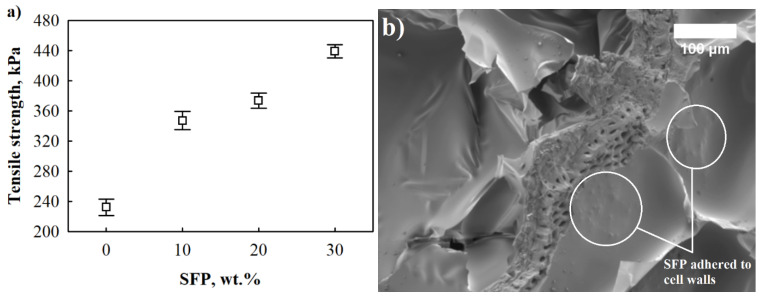
Tensile strength of SFP filler-modified PUR foams and SFP filler interaction with polyurethane foam matrix: (**a**) tensile strength; (**b**) SFP filler particle after tensile strength test (×200 magnification).

The tensile strength test of the SFP filled PUR foams was conducted to investigate the PUR-SFP interaction. The interface between filler and matrix plays an important role in the mechanical properties of all composite materials because the load transfer to the dispersed phase occurs at the interface [45]. The results of SFP filler-modified PUR foams can be observed from Figure 6. It can be clearly seen that the addition of up to 30 wt.% SFP improves the tensile strength of modified PUR foams. The incorporation of 10 wt.% SFP shows a 49% higher improvement of tensile strength compared to neat PUR foam while at 20 wt.% and 30 wt.%, and the tensile strength increased by 61% and 89%, respectively. It is clearly visible that SFP filler particles are attached to the cell walls. Some of them are also detectable in the cell void and a coarse surface can be seen in the cell struts (Figure 6b).

As a result, the improvements are attributed to three fundamental reasons concluded in the study conducted by [46]. The first reason is that, during tensile loading, the filler particles in the PUR foams improve the restriction of the mobility of polymeric chains [47]. The second reason is that the addition of SFP filler to PUR foams helps to increase the resistive surface area which, consequently, increases the capacity of tensile loading in SFP-filled PUR foams [48]. The third reason is that at lower filler contents, the interaction between PUR foam and SFP filler is more effective, and it forms a high matrix-filler reinforcement to resist loading force [49]. These three reasons explain the improvement which is shown in Figure 6a.

**Figure 7:**
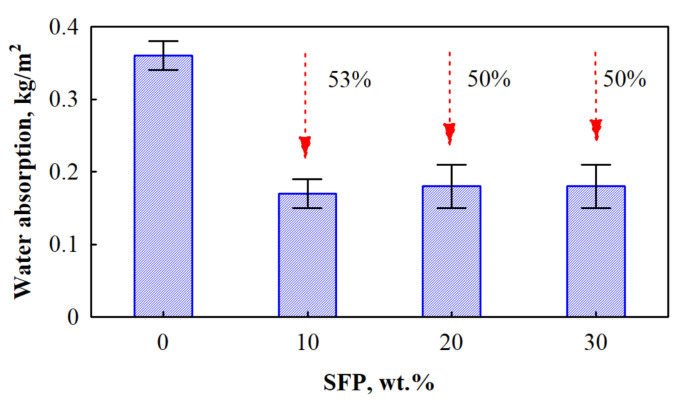
Short-term water absorption by partial immersion of SFP filler-modified PUR foams.

Water absorption is a vital property for materials which will have direct or indirect exposure to water. Short-term water absorption by partial immersion was chosen according to the requirements of the harmonised factory-made PUR products standard [22] and the results are presented in Figure 7.

As the results show, SFP filler considerably improves water absorption of the resulting PUR foams. Interestingly, the reduction is two times higher compared to the neat PUR foam. Firstly, as sunflower press cake is a concentrated fodder which is obtained by pressing sunflower oil, the remaining oil (1.1–5.9 wt.% [50]) in SFP filler acts as a barrier for water to penetrate the particles. Secondly, the tensile strength results (Figure 6) show that the filler-matrix interaction is sufficient and no voids or cracks form at the interface and around the contact zone of the SFP filler and PUR foam matrix. Lastly, the reduction in water absorption may be attributed to an increased closed cell content of SFP filler modified PUR foams, as well (Table 3). The obtained short-term absorption values also indicate that SFP filler-modified PUR foams are competitive with the ones that already exist in the market (up to 0.3 kg/m^2^).

## 4. Conclusions

Polyurethane foam was modified with 10–30 wt.% sunflower press cake filler. The impact of sunflower press cake filler on selected properties of PUR foams, such as rheology (dynamic viscosity, characteristic foaming times), thermal insulating properties (thermal conductivity), mechanical properties (tensile strength and compressive strength perpendicular and parallel to the foaming directions) and water resistance (short-term water absorption by partial immersion) were investigated. It was determined that the greatest improvement was achieved for PUR foams with 20 wt.% SFP. The thermal conductivity was reduced by 17% at an average apparent density value of 65 kg/m^3^, while short-term water absorption by partial immersion results showed that the parameter was twice reduced at 20 wt.% SFP. Mechanical performance was significantly improved; compressive strength perpendicular to the foaming direction increased by 28%, compressive strength parallel to foaming direction increased by 67%, and tensile strength increased by 61%. However, among other properties, significant improvement in mechanical performance was achieved for PUR foams with 30 wt.%; the compressive strength increased by 114% and 71% in the perpendicular and parallel directions, respectively, and tensile strength by 89%.

In summarising the obtained results for SFP filler-modified PUR foams, it is worth noting that such building materials are promising for future application as thermal insulating layers in building envelopes. In addition, the replacement of petroleum-based polyols with plant-based polyols, traditional blowing agents with water, and synthetic fillers with natural or plant-based fillers will contribute to the achievement of sustainability goals and the reduction of carbon dioxide emissions coming from the building industry. However, further studies on plant-based PUR foams are needed in order to reduce thermal conductivity values.

## Figures and Tables

**Table 1:** Formulations of PUR foam and SFP filler-modified PUR foams.

Raw Material	Content, Pbw ^1^
BioPolyol RD	40
Petol PZ 400-4G	60
Water (distilled)	2.7
Polycat 9 (catalyst)	1
ST-52 (surfactant)	3
SFP filler, wt.%	0; 10; 20; 30
Isocyanate index	1.25

^1^ Parts by total weight of polyols.

**Table 2:** Parameters of the foaming mixtures.

Parameter	SFP Filler, wt.%			
	0	10	20	30
Dynamic viscosity mPa·s	115 ± 5	130 ± 3	275 ± 4	400 ± 6
Cream time, s	25 ± 2	31 ± 3	40 ± 4	53 ± 3
Gel time, s	53 ± 3	63 ± 2	72 ± 3	89 ± 3
Tack-free time, s	76 ± 2	96 ± 3	112 ± 2	136 ± 3

**Table 3:** Structural parameters of SFP filler-modified PUR foams.

Parameter	Amount of SFP Filler, wt.%			
	0	10	20	30
Average cell size, μm	566 ± 12	516 ± 24	364 ± 15	272 ± 22
Closed cell content, vol.%	81 ± 2	87 ± 2	90 ± 1	85 ± 4

## Data Availability

Data sharing not applicable.
